# Monitoring long-lasting insecticidal net (LLIN) durability to validate net serviceable life assumptions, in Rwanda

**DOI:** 10.1186/1475-2875-13-344

**Published:** 2014-09-01

**Authors:** Emmanuel Hakizimana, Beatus Cyubahiro, Alphonse Rukundo, Allan Kabayiza, Alphonse Mutabazi, Raymond Beach, Roopal Patel, Jon E Tongren, Corine Karema

**Affiliations:** Malaria and other Parasitic Diseases Division/Rwanda Biomedical Center, Ministry of Health, Kigali, Rwanda; Entomology Branch, Division of Parasitic Diseases and Malaria, Center for Global Health, Centers for Disease Control and Prevention, Atlanta, GA USA; President’s Malaria Initiative (Centers for Disease Control), United States Agency for International Development, Kigali, Rwanda; Laboratory of Entomology, Wageningen University, Wageningen, The Netherlands

**Keywords:** LLIN, Serviceable life, Durability, Survivorship/attrition, Fabric integrity, Loss, Distribution/replacement time

## Abstract

**Background:**

To validate assumptions about the length of the distribution–replacement cycle for long-lasting insecticidal nets (LLINs) in Rwanda, the Malaria and other Parasitic Diseases Division, Rwanda Ministry of Health, used World Health Organization methods to independently confirm the three-year LLIN serviceable life span recommendation of WHO.

**Methods:**

Approximately 3,000 coded LLINs, distributed as part of a national campaign, were monitored in six sites, by means of six–monthly visits to selected houses. Two indicators, survivorship/attrition, a measure of the number of nets remaining, and fabric integrity, the proportion of remaining nets in either ‘good’, ‘serviceable’ or ‘needs replacement’ condition, based on holes in the net material, were tracked. To validate the assumption that the intervention would remain effective for three years, LLIN coverage, calculated using either survivorship, or integrity, by removing nets in the ‘needs replacement’ category from the survivorship total, was compared with the predicted proportion of nets remaining, derived from a net loss model, that assumes an LLIN serviceable life of three years.

**Results:**

After two years, there was close agreement between estimated LLIN survivorship at all sites, 75% (range 64-84%), and the predicted proportion of nets remaining, 75%. However, when integrity was considered, observed survivorship at all sites, declined to 42% (range 10-54%).

**Conclusions:**

More than half, 58%, of the LLINs fell into the ‘needs replacement’ category after two years. While these nets were counted for survivorship, they were judged to be of little-to-no benefit to a user. Therefore, when integrity was taken into account, survivorship was significantly lower than predicted, suggesting that net serviceable life was actually closer to two, rather than three years, and, by extension, that the impact of the intervention during year three of the LLIN distribution-replacement cycle could be well below that seen in years one and two.

## Background

Large-scale distribution of LLINs to achieve universal net coverage has been associated with highly successful malaria control outcomes in Rwanda. When insecticide-treated net ownership increased from 15% to 82%, alongside scale-up of other malaria control interventions, there was a 50% decrease in all-cause mortality of children less than five years old [1]. Building on this success, Rwanda became one of the first countries in Africa to achieve universal LLIN coverage by distributing over 6.1 million LLINs (2010–2011).

In addition to achieving high coverage, the Rwanda LLIN distribution programme is also focused on timely replacement of existing nets. That is maximizing the time between distribution and replacement (for optimum use of resources), while, at the same time, avoiding loss of impact, associated with net failure. Based on LLIN durability monitoring norms [2–5], the National Malaria Control Programme (NMCP), in charge of LLIN distribution and replacement, assumes that nets last three years, and that the proportion of nets remaining at any given time is predicted by a three-year NetCALC net loss model [6]. This report describes monitoring carried out to validate this assumption.

LLIN durability monitoring focuses on three indicators: net survivorship, an estimate of coverage, that is, the percentage of nets still present and in use in the house hold to which they were distributed; fabric integrity, a quantification of the number and size of holes in the LLIN netting and bio-efficacy, a measure of net insecticidal effect. The term ‘hole’ is used as a general term to describe all damage: tears, burn holes, rodent (chewing)-associated damage, rips in corners and seams. While all three indicators are assessed in Rwanda, two: survivorship and fabric integrity are discussed here. The two should be assessed together, because if a net is present and counted for survivorship, but in such poor physical condition that it offers the user little-to-no protection, then survivorship data alone will, most likely, under estimate net loss. This report presents the results from a prospective, longitudinal assessment of LLIN durability, undertaken by the Malaria and Other Parasitic Diseases Division (MAL & OPDD) to monitor both survivorship and fabric integrity.

## Methods

### Site selection

**Figure 1:**
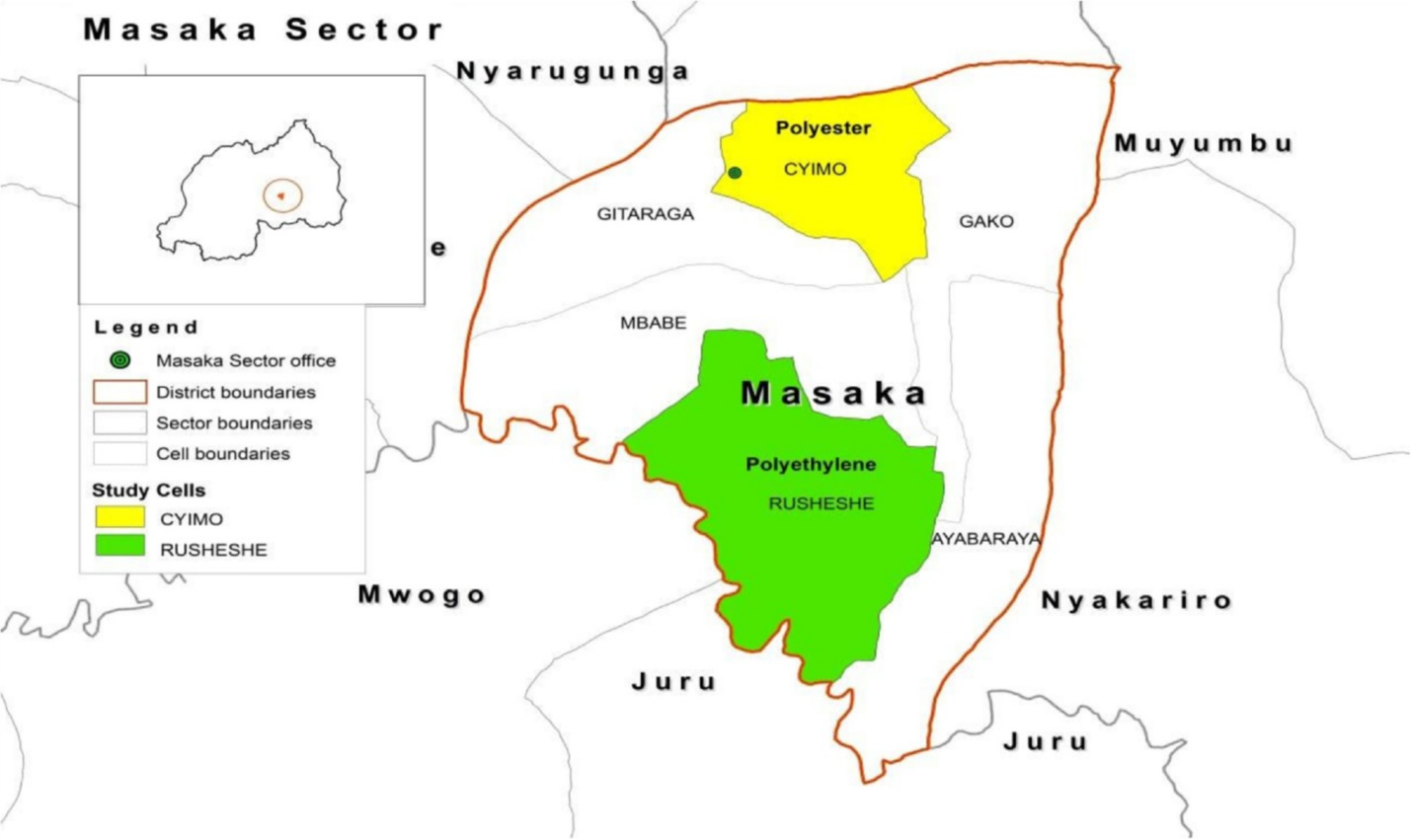
**Peri**-**urban LLIN durability monitoring sites.**

**Figure 2:**
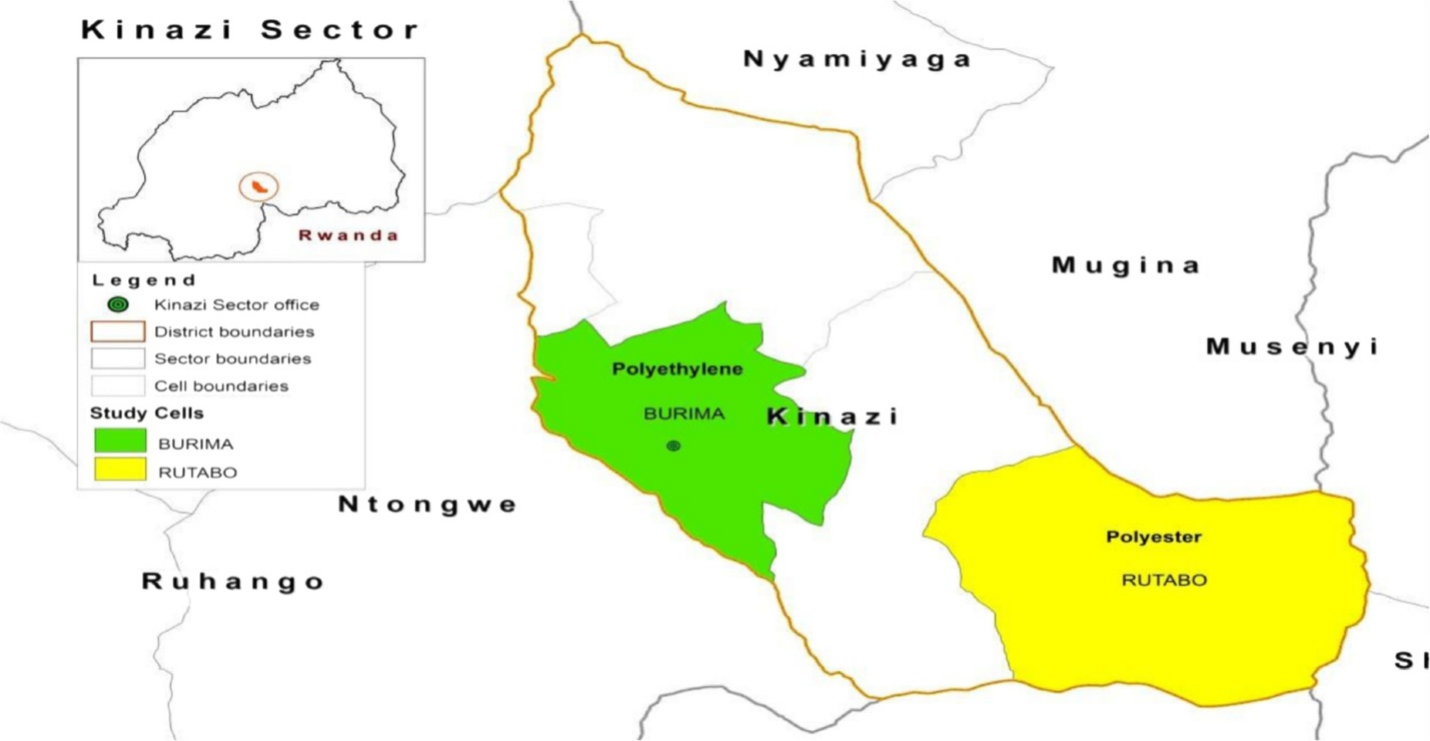
**Rural LLIN durability monitoring sites (endemic transmission).**

**Figure 3:**
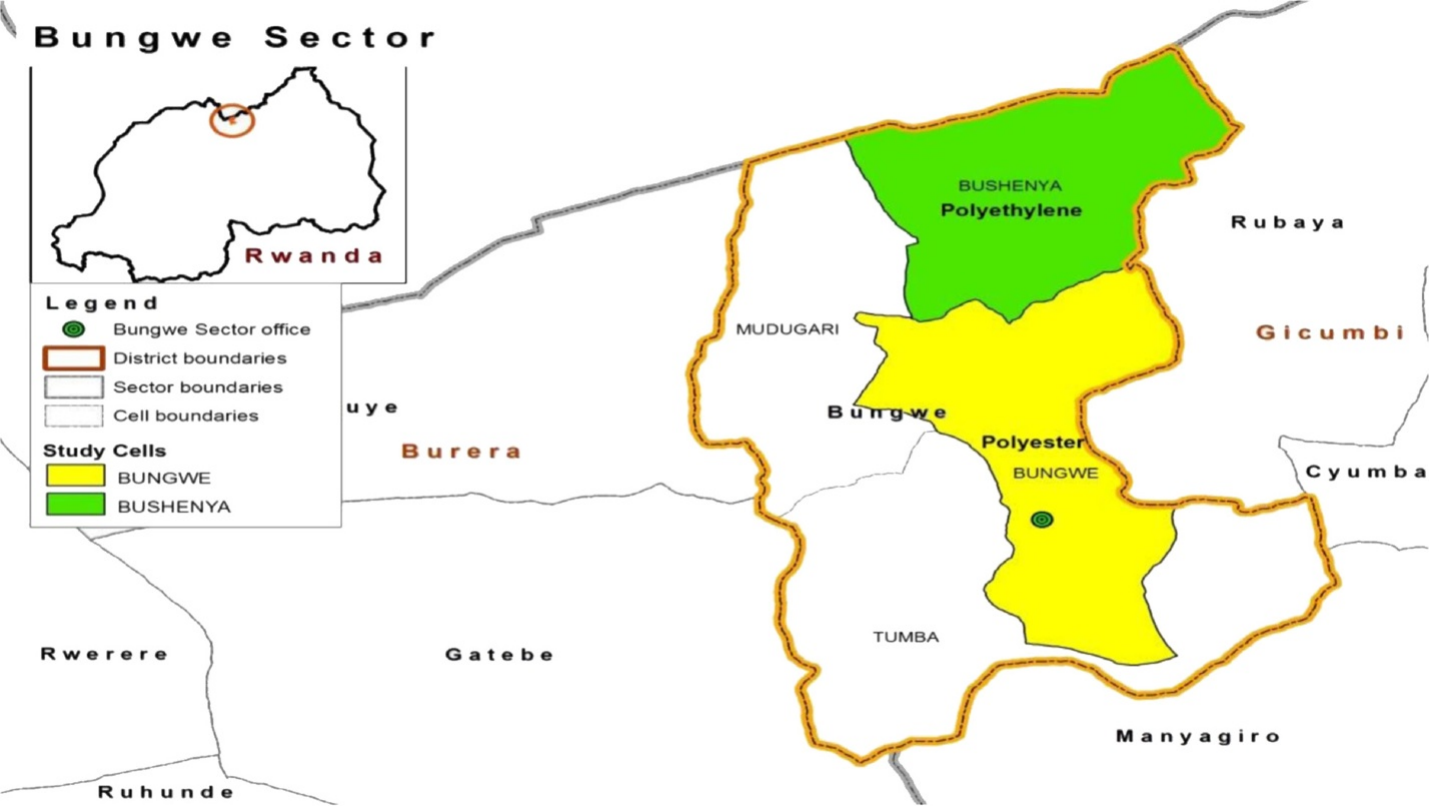
**Rural LLIN durability monitoring sites (hypo-endemic transmission).**

Monitoring was based on a prospective longitudinal assessment, with six-monthly follow up, of six LLIN cohorts. Administrative units, cells (the Rwanda Constitution divides the country into provinces, districts, cities, municipalities, towns, sectors and *cells*, with borders established by Parliament) containing approximately 500 households, selected by probability sampling, were used as monitoring sites. One LLIN per household at the selected sites was followed. Sites in three settings were included in the assessment: a peri-urban setting with endemic malaria transmission, a rural setting with hypo-endemic transmission, and a rural setting with endemic transmission. Peri-urban sites adjoin urban areas, but are localized outside formal urban boundaries and jurisdictions; such sites are becoming urbanized, and progressively assume many of the characteristics of urban areas. Net distribution was implemented such that one of the two sites in each setting received LLINs, manufactured with polyethylene thread while the other site received LLINs manufactured with polyester thread. Table 1 summarizes relevant information about the LLIN monitoring sites. Maps (Figures 1, 2 and 3) give the geographic location within the country.

**Table 1:** **LLIN durability monitoring sites: characteristics (sector,** ***setting*** *), cell, thread (polyethylene or polyester)** and tagging information***

Characteristics		Thread	
Sector (setting*)	Cell	Polyethylene**	Polyester**
Masaka (peri-urban, endemic*)	Rusheshe	500 nets	
		Ink code: one black dot	
		Bar codes A001-A500	
	Cyimo		500 nets
			Ink code: two black dots
			Bar codes B001-B500
Kinazi (rural, endemic*)	Burima	500 nets	
		Ink code: one red dot	
		Bar codes C001-C500	
	Rutabo		500 nets
			Ink code: two red dots
			Bar codes D001-D500
Bungwe (rural, hypo-endemic*)	Bushenye	500 nets	
		Ink code: one green dot	
		Bar codes E001-E500	
	Bungwe		500 nets
			Ink code: two green dots
			Bar codes F001-F500

*endemic* malaria transmission ‘constant’.

*hypo*-*endemic* malaria transmission ‘sporadic’.

^**^polyester thread - denier 100, polyethylene thread - denier 150.

^***^Each LLIN enrolled in the tracking assessment was bar coded. A second site-specific ink code, one or two colored (black, red or green) dot(s), 0.5 cm in diameter, drawn with indelible-ink laundry marker, was also used.

### LLIN distribution and baseline data collection

LLINs were distributed to the selected sites as part of a 2010, countrywide, mass net distribution campaign. After one month, LLIN tracking teams visited 500 houses at each site. If the house was open and at least one campaign net was in use, the head of the household was asked to approve participation in the assessment, which included four follow up visits at six (T_6_), 12 (T_12_), 18 (T_18_), and 24 (T_24_) months post distribution. Upon approval, one of the recently distributed LLINs/household was bar and color coded (Table 1), and the house, to which the net was distributed, was enrolled in the assessment. There was an initial (T_0_) inspection of LLIN fabric integrity, and enrolled households were geo-referenced to facilitate follow up visits. Table 2 summarizes the status of the assessment LLINs at T_0_.

**Table 2:** **LLIN durability: Survivorship (%) at one month post distribution (T**_**0**_ **)**

Setting	Cell	Thread	House-holds visited dropped ^*^		LLINs (households) enrolled ^**^	% LLIN survivorship ^***^ T _0_
Peri-urban/endemic	Cyimo	Polyester	500	93	407	100
	Rusheshe	Polyethylene	500	32	468	
Rural/hypo- endemic	Bungwe	Polyester	500	24	476	
	Bushenya	Polyethylene	500	17	483	
Rural/endemic	Rutabo	Polyester	500	63	437	
	Burima	Polyethylene	500	26	474	

^*^Households were dropped at T_0_ because they were either closed, or open, but had not received an.

LLIN during the national campaign.

^**^Households re-visited during T_6_ follow up visit.

^***^By definition, the percentage (%) of enrolled households with a coded LLIN at T_0_ is 100%.

### Data measurement and estimation of coverage and integrity

During six-monthly follow up visits, T_6_, T_12_, T_18_, and T_24_, all households, open for inspection, were re-visited to assess LLIN survivorship, which was estimated using the equation:


Fabric integrity was also assessed during each visit. The condition of all nets was derived from an examination of 30 assessment LLINs per site. The nets, randomly-selected from all households inspected, were examined for holes and a pHI, the weighted summary of observed damage (holes), was calculated. In this study a method described at a WHO Vector Control Working Group (VCWG) work stream [7] was used to calculate pHI. The LLINs were temporarily removed from houses, fitted over a frame, and subjected to a side-by-side, plus top, visual examination. Technicians recorded the number and size (length) of each observed hole. Based on the result each hole was assigned to one of four shape/size categories:

1. A circle with an estimated diameter of 0.5-2 cm (for a hole judged to be ’smaller than one’s thumb’)
2. A circle with an estimated diameter of 2–10 cm (for a hole judged to be ’larger than one’s thumb, but smaller than one’s fist’)
3. A circle with an estimated diameter of 10–25 cm (for a hole judged to be ’larger than one’s fist but smaller than one’s head’)
4. A circle with an estimated diameter of >25 cm (for a hole judged to be ’larger than one’s head’)

To calculate the pHI, the number of holes in each category was multiplied by a category weight: 1 for category (1), 23 for category (2), 196 for category (3) and 578 for category (4).

Integrity data were entered into the following formula to estimate a pHI for each LLIN:

pHI = (1) (number of category 1 holes) + (23) (number of category 2 holes) + (196) (number of category 3 holes) + 578 (number of category 4 holes).

PHI thresholds [8], were used to ‘translate’ observed pHI results into three integrity ‘condition’ categories:

1. A net with a pHI < 64 was classified as being ‘in good condition’.
2. A net in the 64 ≤ pHI ≤ 768 range, was classified as being ‘in serviceable condition’ (repairable).
3. A net with a pHI > 768, was judged to be ’in need of replacement’ and of questionable benefit to user’ [8].

The estimated median size of a single hole corresponding to each category is:

- 1.6 cm^2^ for category (1) - good
- 168 cm^2^ for category (2) - serviceable
- 1,190 cm^2^ for category (3) - replace

To express the impact of change in fabric integrity on survivorship (net loss), the estimated number of nets in category 3 was removed, and survivorship was recalculated based on the number of nets in categories 1 and 2 only.

Estimates of survivorship, based on coverage and integrity, are shown in tabular form, and are plotted against time for comparison with the NetCALC -predicted proportion of nets, based on a three-year serviceable life assumption.

### Study clearance

This observational study was planned with, and approved by, the Ministry of Health. Community leaders were consulted before the study began, and all gave verbal consent in advance. Head of households gave their written consent prior to being enrolled.

## Results

### Survivorship/attrition

On average, LLIN survivorship declined to a mean of 75% (all sites), range 64-84%, after two years (Table 3). There was a small but significant difference between survivorship of polyethylene and polyester LLINs at the peri-urban sites, 84% versus 64% (*p* < *0.05*), and at the rural, hypo-endemic sites, 78% versus 70% (*p* < *0.05*). While the same pattern was seen at the rural, endemic sites, 79% versus 77%, the difference was not significant (*p* > *0.05*).

**Table 3:** LLIN durability: Survivorship (%), observed and predicted*, by cell, setting, and LLIN thread type at one (T_**0**_ **), 12 (T**_**12**_ **) and 24 (T**_**24**_ **) months post distribution**

Cell	Setting	Thread	Observed			Predicted*
			T _0_	T _12_	T _24_	
Cyimo	Peri-urban	Polyester	100	89	64^1^	75
Rusheshe	Endemic	Polyethylene	100	93	84^1^	
Bungwe	Rural	Polyester	100	92	77	
Bushenya	Endemic	Polyethylene	100	94	79	
Rutabo	Rural	Polyester	100	95	70^2^	
Burima	Hypo-endemic	Polyethylene	100	90	78^2^	

^1,2^ *p* < *0.05.*

*NetCALC predicted percent of nets remaining after two years, based on a 3-year serviceable life assumption.

**Figure 4:**
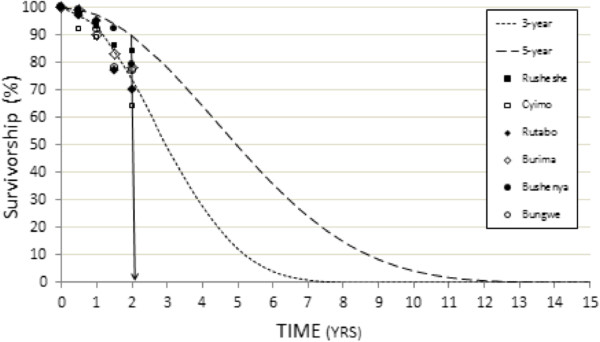
**LLIN observed survivorship by site (data from Table** 3 **with addition of 6-, and 18-month results) versus NetCALC-predicted ‘proportion of LLIN’s present’ assuming either a 3-year (small-dash curve) or a 5-year (larger-dash curve) LLIN serviceable life.**

Figure 4 presents the data from Table 3, as well as T_6,_ and T_18_ survivorship results. NetCALC curves, showing (predicted) proportion of surviving LLINs, based on either a three-year or a five-year net replacement cycle (serviceable life assumption) are shown as dotted lines for comparison.

In summary, observed survivorship at most of the assessment sites appear to track the NetCALC curve based on a three-year serviceable life assumption.

### Fabric integrity

Fabric integrity results (Table 4) are presented by site and thread type, based on the three integrity condition categories: ‘good’ , ‘serviceable’ and ‘replace’ , at baseline and after one (T_12_) and two (T_24_) years. After two years, an estimated 77% of the remaining LLINs in the peri-urban sites (Cyimo and Rusheshe) *versus* 49% of the LLINs in the rural sites (Bungwe, Bushenya, Rutabo, and Burima) fell into the ‘replace’ category. However, of greater interest than site specific differences in integrity, was the fact that after two years, an estimated 47% to as many as 90% of remaining LLINs fell into the ‘replacement’ category.

**Table 4:** LLIN durability: Fabric Integrity expressed as percentage of LLINs in one of three fabric integrity categories*: good, serviceable or needs replacement (replace) by cell and LLIN thread type at T_**0**_ **, T**_**12**_ **, and T**_**24**_

Cell	Cyimo	Rusheshe	Bungwe	Bushenya	Rutabo	Burima
LLIN thread	p-ester	p-ethylene	p-ester	p-ethylene	p-ester	p-ethylene
**T**_**0**_						
Good	100	100	100	100	100	100
Serviceable	0	0	0	0	0	0
Replace	0	0	0	0	0	0
**T**_**12**_						
Good	47	20	67	37	27	60
Serviceable	20	43	13	13	37	27
Replace	33	37	20	50	13	13
**T**_**24**_						
Good	10	3	3	3	10	6
Serviceable	27	7	47	50	37	47
Replace	63	90	50	47	53	47

**Figure 5:**
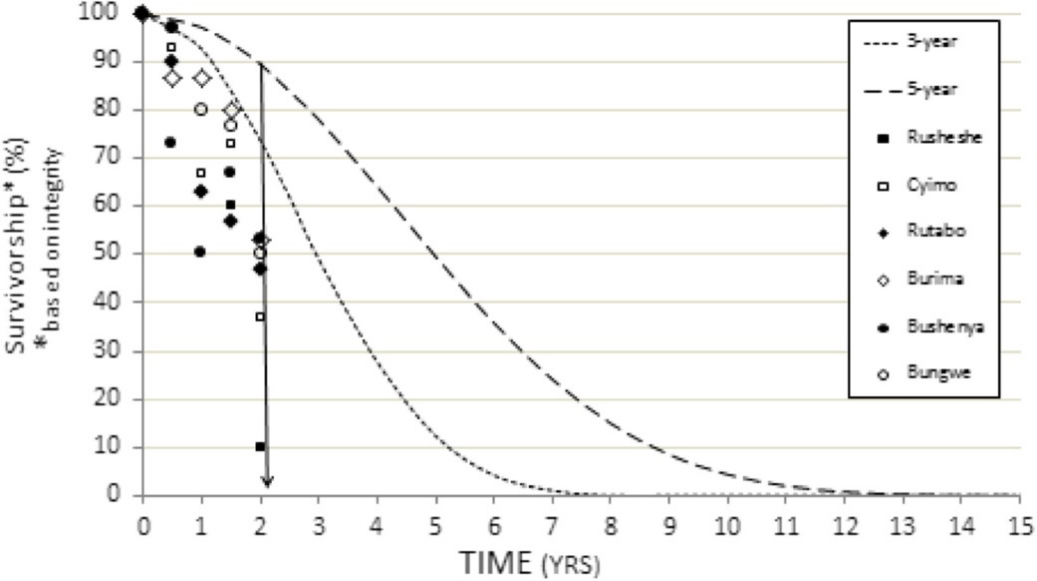
**LLIN observed integrity (data from Table** 4 **expressed as survivorship) versus NetCALC-predicted ‘proportion of LLINs present’ assuming either a 3-year or a 5-year LLIN replacement cycle.** The vertical line bisecting the two year time point (x-axis) facilitates comparison of observed and predicted survivorship.

When the T_24_ integrity measurements were converted to survivorship, by discounting LLINs in the ‘replace’ category, the resulting survivorship estimates (mean values) were between 21 and 65 percentage points below the 75% value predicted by the 3-year serviceable life model (Figure 5).

## Discussion

LLIN durability monitoring in Rwanda indicated lower survivorship, *viz*. greater than anticipated net loss, associated with poor fabric integrity, during year two of a three year LLIN distribution-replacement cycle. The proportion of the remaining nets in need of replacement, after two years, was large enough to suggest that the intervention would lose impact during year three of the distribution-replacement cycle.

Karema *et al*. [1] suggests low net (ITN) coverage (*viz*. survivorship), as one explanation for a 2009 resurgence of malaria in Rwanda following a 2006 under-five, country-wide bed net campaign. The present results highlight an additional possibility: that of greater than expected net loss associated with poor fabric integrity. In the present study, coverage, based on survivorship, decreases by 26% to 36% by the end of year two (post-distribution). However, it is the striking loss of fabric integrity, in addition to reduced coverage, that strengthens the argument that LLIN serviceable life and, by extension, the effective life of an LLIN intervention was, perhaps, one year less than the assumed duration of the LLIN distribution – replacement cycle.

In this analysis, a ‘missing’ net reduces survivorship. However, such ‘missing’ nets could have been present in another house, not visited by the tracking team, and, therefore, still have contributed to coverage and impact. On the other hand, the poor fabric integrity results after two years, suggest that, regardless of whether or not a net was present and in use somewhere in the community, it was as likely to be torn, in need of replacement, and of little use to the user, as to be in good or serviceable condition.

When poor fabric integrity results for surviving nets are considered, survivorship after two years is nearly one half of that predicted by a NetCALC model (41% versus 75%). The assumption that most of these damaged nets probably provided ‘questionable benefit to users’ [8], raises a red flag with respect to the programmatic assumption that the LLIN intervention would remain effective during year three of the distribution replacement cycle.

NetCALC curves, based on a three-to-five year serviceable life, are thought to be realistic estimates for programme planning. However, the results reported here, suggest that many, perhaps most of LLINs, in use in Rwanda, are of questionable benefit to users, due to poor fabric integrity, after two years. If one assumes that such nets, more appropriately, belong in the nets lost (associated with survivorship) category, then LLIN survivorship estimates decrease dramatically. When such results are compared with expected survivorship, derived from NetCALC predictions, the discrepancy is significant.

Factors that affect LLIN durability, act to a greater or lesser extent, in different settings. For example, in the present assessment, the rate at which holes appear (loss of fabric integrity) in nets is higher in urban locations. Given the number of factors that affect LLIN durability, and the variation between settings where they are distributed, it is not surprising that reliance on generalizations about how long nets last could be misleading. LLIN monitoring based on the approach outlined by WHO, is recommended as the best way to address the programmatic question of how long nets last.

If faster-than-expected fabric degradation is a significant determinant of LLIN impact, what are the possible ways to ameliorate its effect? Unfortunately, reducing the time to LLIN replacement is problematic, based on current funding models (nonetheless, the Ministry of Health, Rwanda is considering LLIN replacement every 30 months, versus the current 36–40 months). Other alternatives: working with manufacturers to develop and test more durable net products, fostering a stronger net care and repair culture, and ‘pushing’ more nets to communities via routine channeling and social marketing, as well as national campaigns, are also planned to close the gap between observed and expected net loss rates. Finally, as stated earlier, a comprehensive programme of LLIN durability monitoring to reduce reliance on assumptions, that may not apply everywhere, is needed to accurately inform programme about ‘on-the-ground’ net loss rates.

When LLINs remain in use after ‘failing’ due to loss of fabric integrity, communities, where LLIN coverage, as well as compliance with nightly net use, appears to be adequate, may still experience a resurgence of malaria. For this reason, indicators of LLIN coverage, based on questions such as “did you sleep under a bed net last night?” [9], should not, necessarily, be interpreted as a confirmation of protection from malaria transmission.

This study did not address the third WHO durability indicator, bio-efficacy. Could, for example, insecticidal effect compensate for poor fabric integrity? Unpublished data from Rwanda suggests that after two years, when net loss associated with fabric integrity has taken its toll, LLIN bio-efficacy also declines, permitting some vector survival following exposure to nets. As vector survival increases, vector entry via holes, man-vector contact and transmission rates also increase [10]. The added effect of vector-pyrethroid resistance, the ability of vectors to navigate through holes in nets, and the observation of living vectors resting inside ‘older’ LLINs, all suggest that bio-efficacy, later in the LLIN distribution replacement cycle, no longer compensates for poor fabric integrity.

It is important to note that the thresholds used to evaluate fabric integrity in this study, e.g. pHI-based fabric integrity condition categories, reflect limited observations and should be thought of as more arbitrary than evidence-based. Therefore, their accuracy with respect to the question of net replacement merits additional evaluation. Nonetheless, what pHI threshold-based monitoring does document, regardless of the interpretation, *viz*. condition category, assigned to each threshold, is the rapid degeneration of fabric integrity during years one and two following distribution in Rwanda. In the present study 0% (T_0_), 28% (T_12_), and 58% (T_24_) of nets were estimated to have crossed the most dangerous pHI threshold, equivalent to approximately one square foot of missing net (pHI equivalent to a single hole of 1,190 cm^2^ = 1.2 ft^2^). While the interpretation of pHI thresholds, should be refined, they currently provide a much needed reference for ‘real time’ evaluation of LLIN interventions, in Rwanda and elsewhere.

## Conclusions

Two years after a national LLIN campaign, as many as three in ten LLINs had been removed from the houses where they were hung at distribution. However, more surprising, was the fact that five to as many as nine of every ten remaining LLINs showed loss of fabric integrity to a degree that called into question their ongoing serviceability, during year three of the planned distribution-replacement cycle. If loss of fabric integrity also means loss of protection from man-vector contact, as assumed, then adjustments to LLIN distribution planning are needed, along with more comprehensive monitoring of LLIN durability. As countries continue to scale-up LLIN coverage, it will be important to have specific information on LLIN durability in a variety of settings. This information should be generated in as short a time frame as possible to ‘inform’ decisions on how best to replace failing LLINs before they compromise the efficacy of the intervention.
